# Aggregation modeling of the influence of pH on the aggregation of variably charged nanoparticles

**DOI:** 10.1038/s41598-021-96798-3

**Published:** 2021-08-30

**Authors:** Yu Xiong, Xinmin Liu, Hailing Xiong

**Affiliations:** 1 College of Computer and Information Science, Southwest University, Chongqing, 400715 China; 2 College of Resource and Environment, Southwest University, Chongqing, 400715 China

**Keywords:** Nanoparticles, Physics

## Abstract

The aggregation of variably charged nanoparticles is usually induced by the changes in internal and external conditions, such as solution temperature, pH, particle size, van der Waals force, and electrostatic repulsion among particles. In order to explore the effect of pH on the aggregation of variable charge nanoparticles, this paper proposed an extended model based on the 3D on-lattice Cluster–Cluster Aggregation (CCA) model. The extended model successfully established the relationship between pH and sticking probability, and used Smoluchowski theory to calculate the aggregation rate of nanoparticles. The simulation results showed that: (1) the change of the aggregation rate of the variable charge nanoparticles with pH conforms to the Gaussian distribution, (2) the initial particle concentration has a significant effect on the aggregation rate of the nanoparticles, and (3) pH can affect the competition between van der Waals force and electrostatic repulsion between particles, thereby affecting the degree of openness of clusters. The research demonstrated the extended CCA model is valuable in studying the aggregation of the variably charged nanoparticles via transforming the corresponding influence factors into the influence on the sticking probability.

## Introduction

The research on the aggregation of particles in the nano and micrometer size range is relevant in numerous processes, such as in the production of chemical materials and medicine, in environmental protection, and so forth^1–4^. The main factors affecting the aggregation of particles: pH^4–7^, solution temperature^7^, ionic strength^8,9^, long-range van der Waals forces^10^, gravity^11,12^, electrolyte concentration^12^, and electrolyte type^13^, particle size, the surface charge density of particles, etc. The researches showed that the surface charges of particles is mainly divided into variable and permanent surface charge^5^. The charge of the variably charged surface depends not only on the chemical surface, but also on the composition of the solution. The variably charged surface is a hydrated oxide surface (i.e. M–OH), and the $\hbox {H}^{+}$ in the solution with lower pH can combine with M–OH on the surface of particles, which leads to the increase in positive charges on the surface of particles. On the contrary, the $\hbox {OH}^{-}$ in higher pH solution can induce the dissociation of $\hbox {H}^{+}$ on the surface of particles, resulting in the increase in negative charges on the surface of particles. Therefore, there exists a pH value which makes the surface of the particles uncharged, the pH is called the point of zero charge (PZC) of the variably charged particle^5^. Li et al. proved that only the two DLVO forces, electrostatic and van der Waals forces should be considered in the study of the aggregation of variably charged particles^5,14^. As mentioned above, the electrostatic forces of variably charged particles depend on pH, so the aggregation of variably charged particles would be pH dependent.

It is a great challenge for the researchers to precisely control the dynamic aggregation process in laboratory experiments. The aggregation model bridges the study of particle aggregation by computer simulation and laboratory experiments. There are some common aggregation models, such as the Diffusion-Limited Aggregation (DLA) model, the Reaction-Limited Aggregation (RLA) model, and the Cluster–Cluster Aggregation (CCA) model. Among those models, the Cluster–Cluster Aggregation (CCA) model is considered to be suitable for describing particle aggregation^15,16^. The CCA model takes the Monte Carlo simulation method as the core idea and was divided into Diffusion-Limited Cluster Aggregation (DLCA) model and Reaction-Limited Cluster Aggregation (RLCA) model by the probability of aggregation of particles after a collision, and the probability of aggregation can be expressed as the sticking probability^16–18^. The DLCA model assumes two particles (clusters) can stick together once they collide, which means the sticking probability is 1, which reflects the rapid aggregation. And the RLCA model assumes the two particles (clusters) may not stick together after the collision, but stick together with a sticking probability less than 1, which reflects the slow aggregation. James and Sasikumar^6^ studied the effects of solvent evaporation and pH on the gelation rate and gel structure based on the classic CCA model. However, in their experiments, many simplifications were performed on the evaporation and pH change of the solution, and the pH of the solution was limited to less than or equal to 7. And their research did not directly calculate the gelation rate, instead, the rate of gelation is reflected by the number of clusters in the system at the same time.

In this paper, an extended CCA model was proposed to study the influence of pH on the aggregation of variably charged nanoparticles. In this study, pH is the only variable, and the influence of other factors is not considered. In the extended CCA model, the sticking probability in the classic CCA model was modified as a function related to the point of zero charge (PZC)of variably charged nanoparticles and pH based on the research of J. James and R. Sasikumar. In order to reflect the change of the aggregation rate with pH more completely and accurately, the pH is set as a time parameter to ensure that the pH changes continuously throughout the simulation process. And the aggregation rate was calculated by Smoleuchowski theory. The specific simulation process and results are described as follows.

## Simulation methods

### The diffusion probability

The diffusion process is consistent with the classic CCA model. According to the Stokes–Einstein formula, the initial diffusion coefficient of the single particle can be obtained by^19–22^:

1 $$\begin{aligned} D_0=\frac{KT}{6\pi \mu R_f} \end{aligned}$$

where *K* is the Boltzmann constant, *T* is the absolute temperature, $\mu$ is the viscosity of the solution, and $R_{f}$ is the hydrodynamic radius (larger than the geometric radius) of the single particle^22^. The diffusion coefficient of the clusters consisting of *i* single particles is^19–21^

2 $$\begin{aligned} D_i=D_0S^\gamma \end{aligned}$$

where *S* is the mass of the selected cluster, which is expressed by the number of the single particles contained in the selected cluster, and $\gamma$ is the diffusion exponent. A cluster is randomly selected to diffusion and its diffusion probability $P_{move}$ is calculated by^19–21^:

3 $$\begin{aligned} p_{move}=\frac{D_i}{D_{max}} \end{aligned}$$

where $D_{max}$ is the maximum diffusion coefficient for any clusters in the current system. A random number *X* uniformly distributed in [0,1] is generated and the selected cluster is moved only if $X \le P_{move}$.

### The sticking probability

If a collision occurs between two clusters (one consists of *i* single particles, and another consists of *j* single particles), they can stick together to form a new larger cluster with the sticking probability $P_{ij}$^19–21^:

4 $$\begin{aligned} P_{ij}=P_1(ij)^\sigma \end{aligned}$$

where $P_1$ is the sticking probability of the single particle, $\sigma$ is the sticking probability exponent. $P_{ij}$ is set to 1 if $P_{ij} \ge$ 1. When a collision takes place, a random number *Y* uniformly distributed in [0,1] is generated and compared with the given $P_{ij}$. The collision is considered effective only when $Y \le P_{ij}$ is verified. The sticking probability of particles is set as a function related to pH and PZC in the research of James and Sasikumar^6^:

5 $$\begin{aligned} P_{ij}=P_110^{\mathrm{(pH-PZC)}}. \end{aligned}$$

The PZC was set as 7, and the influence of pH change caused by the evaporation of liquid medium on the particle aggregation rate and gel structure during the gelation process was discussed in the study of James and Sasikumar^6^. Although the difference of the evaporation rate of solute and solvent leads to the fluctuation of pH, the pH will never exceed 7. Therefore, the study did not include the situation when pH is greater than 7. In this paper, pH is the only variable, which means that the surface charge of variably charged particles is only related to pH^5^. So when the pH is equal to PZC, the net surface charge of the particles is zero, which makes the electrostatic repulsion force between the particles zero. At this time, once the particles collide, they will stick together, which corresponds to the rapid aggregation. When the pH is smaller than PZC, the number of positive charges on the surface of the particles will increase. Conversely, when the pH is greater than PZC, the number of negative charges on the surface of the particles will increase. In both cases, the electrostatic repulsion force among particles will increase, which will reduce the sticking probability, and lead to slow aggregation. In the classic CCA model, when $P_{ij}$ = 1, it corresponds to the rapid aggregation, and when $P_{ij}$ < 1, it corresponds to the slow aggregation. According to the above analysis and Eq. (2), the sticking probability of two particles (clusters) after the collision is modified to the relationship related to pH and PZC:

6 $$\begin{aligned} P_{ij}=P_110^{-\mathrm {|pH-PZC|}}. \end{aligned}$$

The modified Eq. (6) can not only reflect the change of the sticking probability when the pH is greater than PZC, but also the change of the sticking probability when pH is less than or equal to the PZC. When the initial sticking probability $P_1$ is set to 1, Eq. (6) includes both the rapid aggregation (pH = PZC) and the slow aggregation (pH $\ne$ PZC). Similarly, a random number *Z* is generated between [0,1]. If $Z \le P_{ij}$, the collision is considered an “effective collision”, which means the two colliding clusters will stick together.

### The aggregation rate of particles

Smoluchowski modeled the aggregation rate of particles by calculating the rate of diffusion of particles toward a central reference particle^23,24^. Du^22^ deduced the theoretical formula for calculating the aggregation rate constant in the rapid aggregation and the slow aggregation of charged particles based on the Smoluchowski theory. In the CCA model, the aggregation process can be regarded as a process in which a moving particle or cluster collides with another static particle or cluster,and aggregates together with a certain probability^25^. According to Smoluchowski theory, the circulation flux of collisions between particles is considered firstly. When the particle *j* is fixed, the flux expression for the particle *i* type of particle that can collide with particle *j* is^22^:

7 $$\begin{aligned} J_i=4\pi R_{ij}D_in_i \end{aligned}$$

where $n_i$ is the concentration of *i* particles or clusters; $R_{ij}$ is the distance between two particles (clusters) when they collide. For a mono-disperse system, $R_{ij}$ is the diameter of the single particles. $D_i$ is the diffusion coefficient of the particle (or cluster) *i*. As mentioned above, particle *j* is fixed, so Eq. (7) is the number of collisions of particles per unit time and unit volume.

For the rapid aggregation, each collision will lead to the aggregation. Therefore, the rapid aggregation rate constant of particles (or clusters) *i* can be deduced as follows:

8 $$\begin{aligned} k_i=4\pi R_{ij}D_i. \end{aligned}$$

According to Eqs. (1) and (2), Eq. (8) can be modified as

9 $$\begin{aligned} k_i=4\pi R_{ij}(\frac{KT}{6\pi R_{f} \mu })S^\gamma \end{aligned}$$

where the value of $\gamma$ (diffusion exponent) can be set to 0 in the CCA model^20^. It is obvious from Eq. (2) when the value of $\gamma$ is 0, the diffusion probability of the selected cluster is independent of the mass of clusters (the number of single particles contained in clusters). Therefore, Eq. (9) can be modified as:

10 $$\begin{aligned} k_i=4\pi (2R)\left(\frac{KT}{6\pi R_{f} \mu }\right)=\frac{4KTR}{3\mu R_{f}}. \end{aligned}$$

Equation (10) represents the formula for the rapid aggregation rate obtained by the Smoluchowski theory combined with the characteristics of the CCA model. For the slow aggregation rate, the particles need to overcome the repulsive barrier after the collision, so the slow aggregation rate can be expressed as^22^:

11 $$\begin{aligned} k_{slow}=\frac{k_i}{W} \end{aligned}$$

where *W* is the stability ratio. According to Eqs. (10) and (11), the slow aggregation rate can be expressed as:

12 $$\begin{aligned} k_{slow}=\frac{k_i}{W}=\frac{4KTR}{3\mu WR_{f}}. \end{aligned}$$

It is obvious from Eqs. (10) and (12), the aggregation rate of particles or clusters is independent of the mass of clusters when the diffusion index is 0. The stability ratio *W* in Eqs. (11) and (12) can be defined as the ratio of “the total number of collisions between particles” to “the effective number of collisions between particles” in the CCA model^26,27^. For the rapid agglomeration, once the particles collide, they can agglomerate together, so the number of collisions between particles is equal to the effective number of collisions, which means $W =1$. For the slow agglomeration, because the repulsion force is considered, most collisions are reflective which enables the particle or cluster to skip from one side to another, and penetrate deeper within the aggregate to make a denser structure^27^, so the number of collisions between particles is always greater than the “effective number of collisions”, that is, $W > 1$. Replacing 1/*W* with $\alpha$ which can be called attachment efficiency, the calculation of $k_{slow}$ can be expressed as:

13 $$\begin{aligned} k_{slow}=\frac{4KT\alpha }{3\mu }\frac{R}{R_{f}}. \end{aligned}$$

In the Eqs. (10) and (12), the *R* (the geometric radius of the single particle) should be smaller than the $R_{f}$ (the hydrodynamic radius of the single particle)^22,28^. Du et al.^22^ proved that the slow aggregation rate calculated by considering the hydrodynamic radius is about 8% lower than the theoretical value, and it has almost no effect on the fast aggregation rate. Therefore, in this paper, it is considered that *R* is equal to $R_{f}$, without considering the specific values of *R* and $R_{f}$ for specific particle types.

## Experimental parameters

As the above discussions, for variably charged particles, the aggregation process depends on pH when the electrolyte concentration, electrolyte type, and temperature of electrolyte solution remain unchanged. To accurately reflect the relationship between aggregation rate and pH, pH was set as a time-dependent parameter in this paper. To simplify, the simulation only considered the influence of pH on particle aggregation and assumed that the electrolyte concentration remained constant throughout the simulation, namely, the ion concentration of the system remained constant. The change of pH was defined as:

14 $$\begin{aligned} pH(t)=vt+pH(t_0) \end{aligned}$$

where *v* is the change rate of pH and pH ($t_0$) is the initial pH (when $t = 0$). The whole simulation was a time-dependent process. After each cluster was randomly selected to move, the simulation time was increased by $\Delta t$^15^:

15 $$\begin{aligned} \Delta t=\frac{1}{N(t)D_{max}} \end{aligned}$$

where *N*(*t*) is the number of clusters (including single particles) in the system at time *t*. However the influence of pH on the diffusion process is not clear, it is assumed that pH has the same effect on the diffusion of clusters of different sizes, so the diffusion index $\gamma$ is set to 0. Referring to the research of James and Sasikumar^6^, the initial sticking probability $P_1$ is set to 1. The spherical single particle diameter $d_1 = 100$ nm, Brownian motion step length $l_B = d_1$, cubic side length $L = 100d_1$ and the initial particle concentration c is set to 0.01, 0.005, 0.0025 (the number of the single particles of the initial system equals to $c*L^3$). The simulation terminates when only one cluster remains in the system or the aggregation rate *k* is 0 (the aggregation no longer occurs in the system).

## Results and discussion

### The influence of pH on the aggregation rate

**Figure 1:**
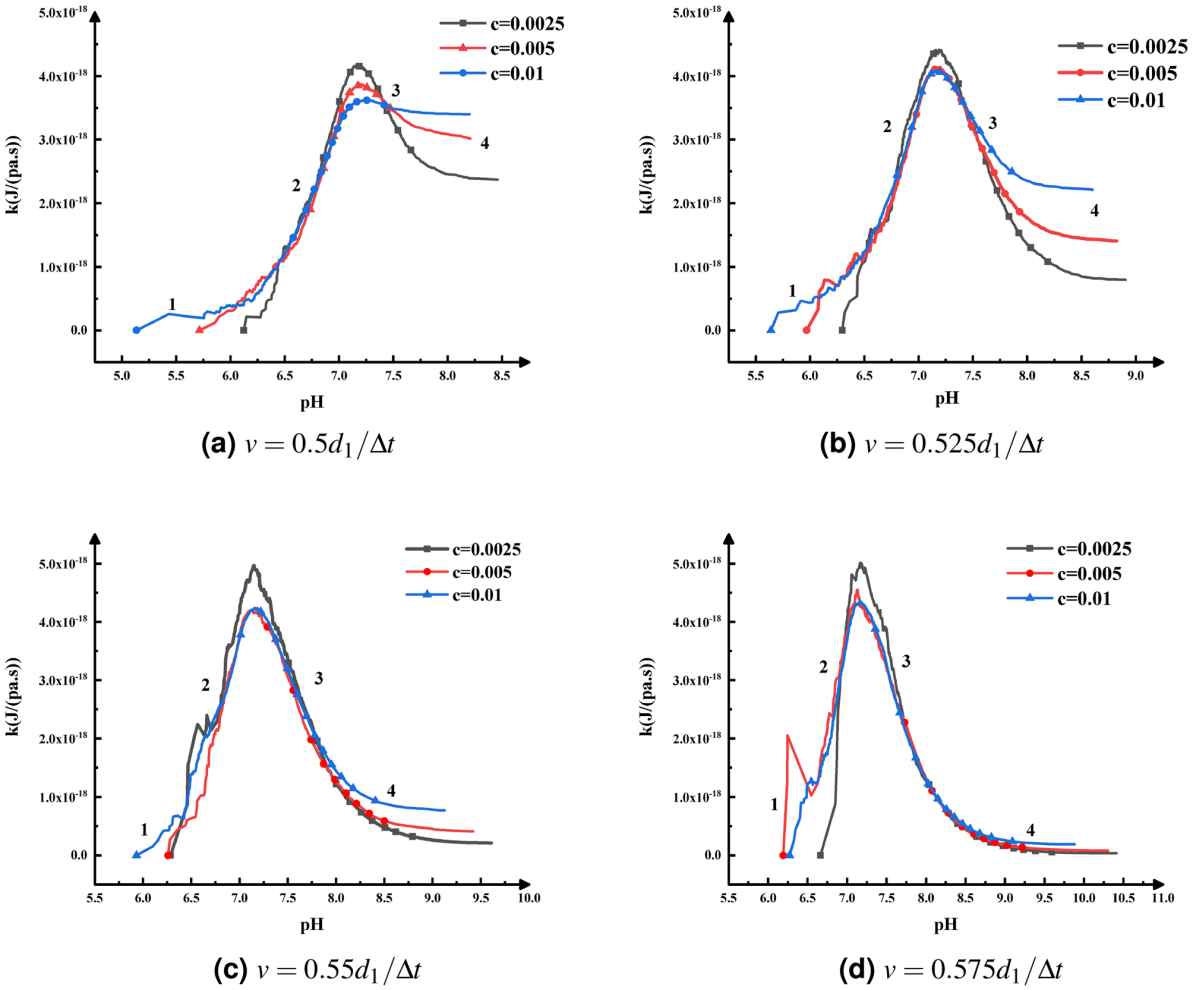
The change of aggregation rate, *k*, with pH for different initial particle concentration and pH change rate.

The changes of aggregation rate with pH at different initial particle concentration and pH change rate are shown in Fig. 1. According to the simulation results (Fig. 1), the changes of particle aggregation rate with pH conform to the Gaussian distribution. The simulation results are consistent with the conclusions obtained by Zhu^5^ through experiments, so the simulation results are reliable. When the pH is infinitely close to PZC, the electrostatic repulsion force between the particles is very small, and may even be zero. Therefore, the aggregation between the particles can be regarded as “rapid aggregation”, so the particles aggregation rate reaches the maximum. When pH increases or decreases along PZC, the surface charge of the variably charged particles increases, resulting in the increase of the electrostatic repulsion force between the particles, which leads to the decrease of the number of the effective collisions. The “slow aggregation” occurs between the particles, so the aggregation rate decreases.

As shown in Fig. 1, the aggregation rate reaches the maximum when pH is always slightly greater than PZC. The main reasons: in the small range where the pH is infinitely close to PZC from the left and right ends, the sticking probability $P_{ij}$ between colliding particles is infinitely close to or equal to 1, and the aggregation can almost be regarded as “rapid aggregation”. In this range, $\alpha$ keeps increasing, and it can be seen from Eq. (13) that the aggregation rate *k* increases in this range. Therefore, the maximum aggregation rate *k* appears when the pH value is slightly higher than PZC. Noticeably, the main purpose of setting different pH change rates is to reflect the change of aggregation rate with pH in more detail and to avoid the case that pH is greater than PZC after the first $\Delta t$.

**Figure 2:**
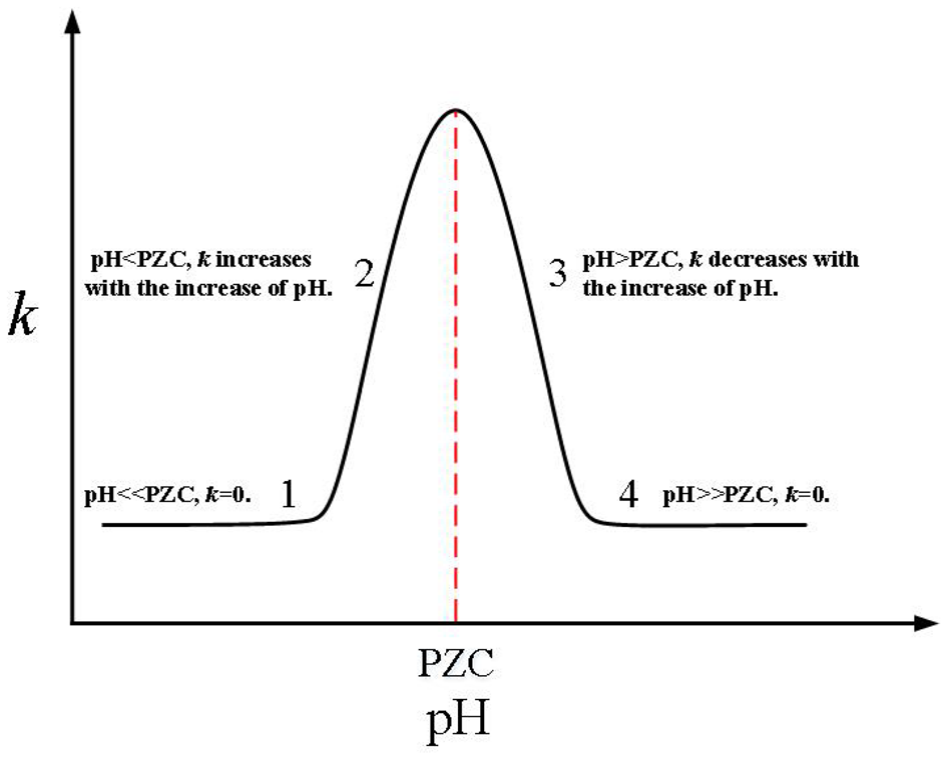
Theoretical prediction diagram of the change of colloidal aggregation rate constant, *k*, with pH^5^.

Compared with the theoretical prediction chart proposed by Zhu (Fig. 2), the change of particles aggregation rate with pH is mainly divided into four stages. The main reason why the “1” stage is not obvious in the simulation results (Fig. 1) is that the simulation time from the beginning ($t = 0$) to the first $\Delta t$ is relatively large, and pH changes rapidly from 0, resulting in the aggregation rate almost growth linearly, so the change process of “1” stage is not obvious. Referring to Eq. (6), when the pH value is far greater or smaller than PZC, the sticking probability particles will be very small. At this time, there is almost no aggregation between particles, so the “1” and “4” stages in Fig. 2 would appear. According to the simulation results (Fig. 1), the aggregation rate changes sharply with pH in the “1” and “2” stages, while the “3” and “4” stages are relatively stable. The possible reason is that in the initial stage of aggregation, the concentration of particles is higher, and there are more collision opportunities between particles, which can increase the number of collisions and effective collisions to a certain extent. However, the sticking probability at this stage is still relatively small. Thus, the conflict among the sticking probability, the number of collisions, and the effective number of collisions leads to the violent fluctuation in the “1” and “2” stages.

**Figure 3:**
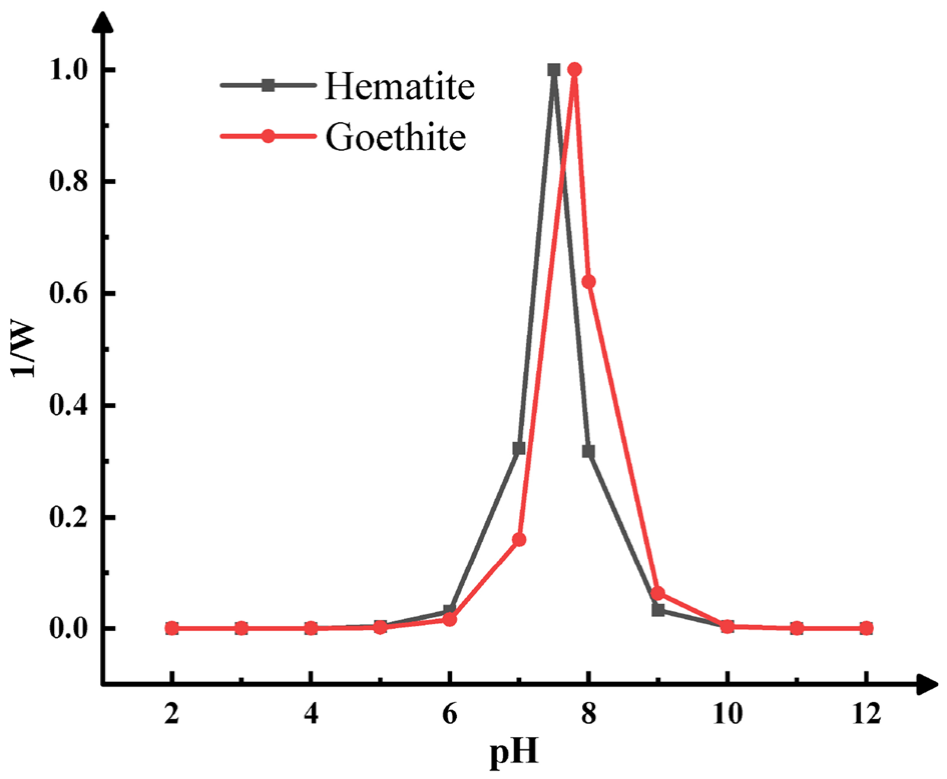
The attachment efficiency (1/*W*) of hematite and goethite nanoparticles in suspensions with different pH obtained by this model.

Through this model, the experiment carried by Xu et al.^13^ in which they studied the effect of pH on the stability of hematite nanoparticles and goethite nanoparticles (PZC of hematite is 7.5, PZC of goethite is 7.8) was reproduced. The relationship of attachment efficiency [in Eqs. (12) and (13)] with pH values is shown in the Fig. 3. It can be found that when the pH of the suspension is less than 6 or greater than 10, the nanoparticle suspension is very stable because the electrostatic repulsion force on the surface of the nanoparticle is much greater than the van der Waals force. When the pH is between 6 and 10, the situation reverses, and aggregation occurs between the nanoparticles. It is obvious from Fig. 3 that when pH is 7.5, the aggregation rate of hematite reaches the maximum, and when pH is 7.8, the aggregation rate of goethite reaches the maximum. The experimental results obtained in this study are basically consistent with those obtained by Xu et al., and are in line with the theory described above. Therefore, the model proposed in this paper can predict the aggregation rate of variably charged nanoparticles and predict the stability pH interval of variably charged nanoparticles.

**Figure 4:**
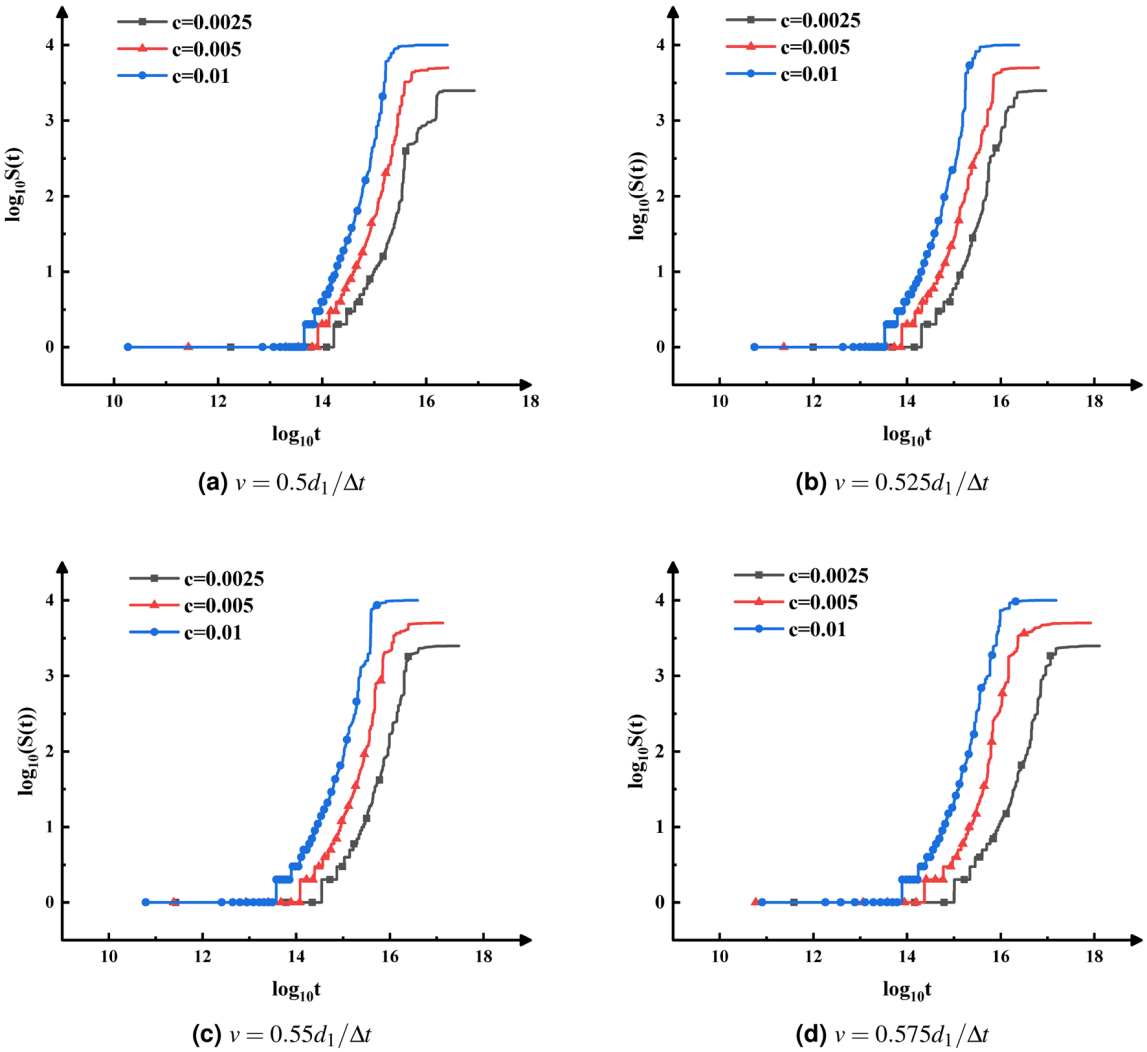
A log–log plot of the weighted average cluster size, *S*(*t*), as a function of time *t*, for different initial particle concentrations and pH.

The changes of the weighted average cluster size with time under different initial particle concentrations and pH change rates are illustrated in Fig. 4. The change of weighted average cluster size can reflect the growth rate of clusters. As shown in Fig. 4, the higher the initial particle concentration is, the earlier the particles start to aggregate and reach a stable state. At the same time and the same pH change rate, the weighted average cluster size increases quickly when the initial particle concentration increases. The results indicated that the effect of initial particle concentration on the aggregation rate cannot be ignored.

### The influence of pH on cluster morphology

**Figure 5:**
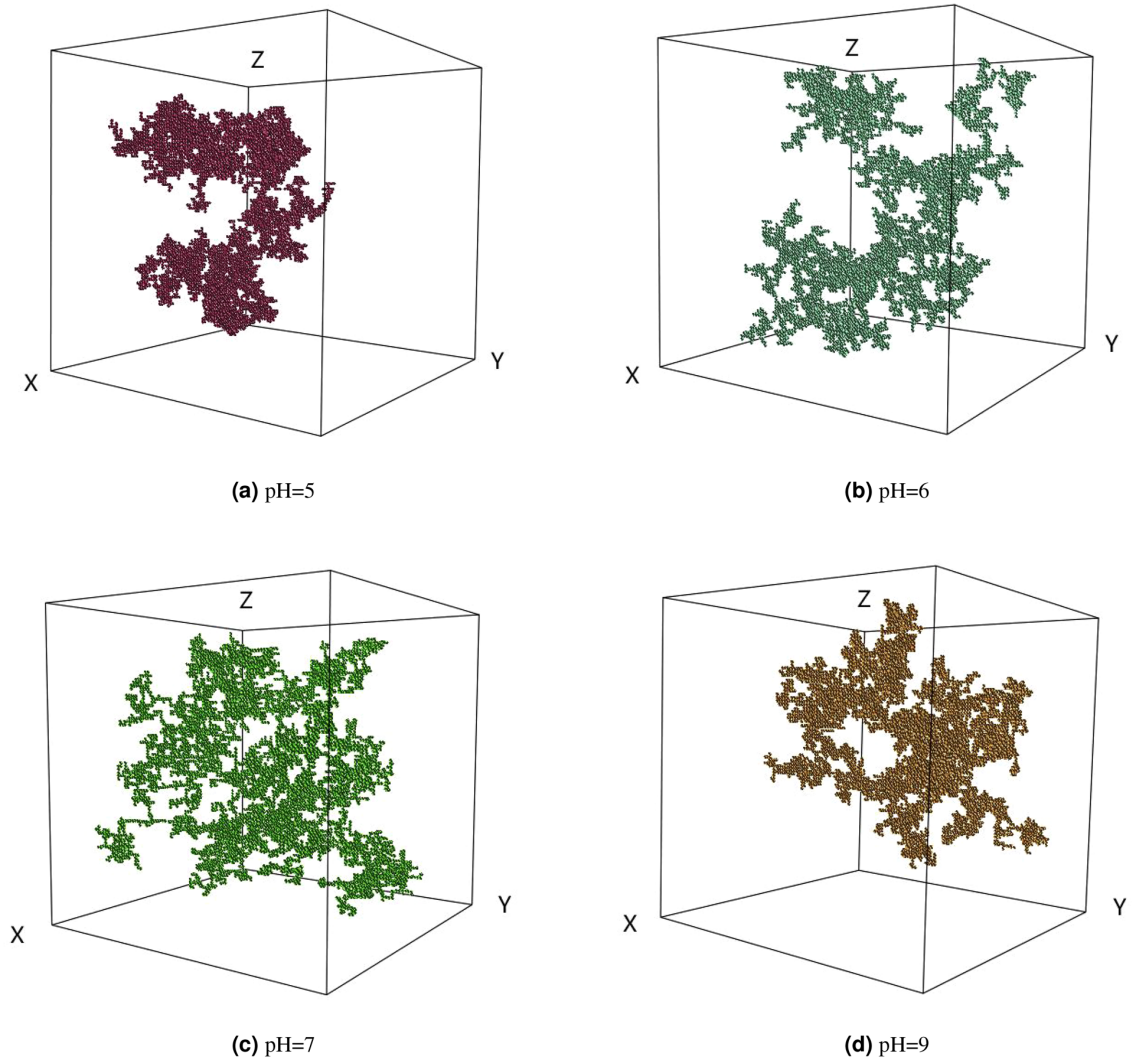
The final cluster structure under different solution pH in the extended 3D on-lattice CCA model.

The final cluster structures under different pH conditions with the initial particle concentration of 0.01 are illustrated in Fig. 5 which were obtained from the computer aggregation model. The fractal dimensions of the clusters in (a) (b) (c) (d) are 1.957, 1.890, 1.776, and 1.943, respectively. The most open cluster structure is observed at pH = 7. When the pH is equal to PZC, the net surface charge of the particles is zero, and there is no electrostatic repulsion force between the particles. And according to Eq. (6), when the pH is equal to PZC, the sticking probability between particles is 1, which corresponds to the rapid aggregation. Once particles collide with each other, they can stick together, thus forming clusters with a relatively open structure and relatively small fractal dimensions(1.776) which is consistent with previous research results^27,29^. When pH is 5 and 9, the net charge on the surface of the particles is not zero, and there is electrostatic repulsion force between the particles. Corresponding to the slow aggregation, it easier for the single particles (or small clusters) to enter the interior of large clusters and form dense clusters^30^. According to Eq. (6), when pH is 5 and 9, the sticking probability is 0.01. However, due to the randomness of the CCA model, the fractal dimensions of clusters generated under the two conditions are slightly different. When pH is 6, the electrostatic repulsion force between the particles is between the values when pH is 5 (or pH = 9) and 7, so the sticking probability and the fractal dimension of the clusters formed are also between the values when pH is 5 (or pH = 9) and 7.

**Figure 6:**
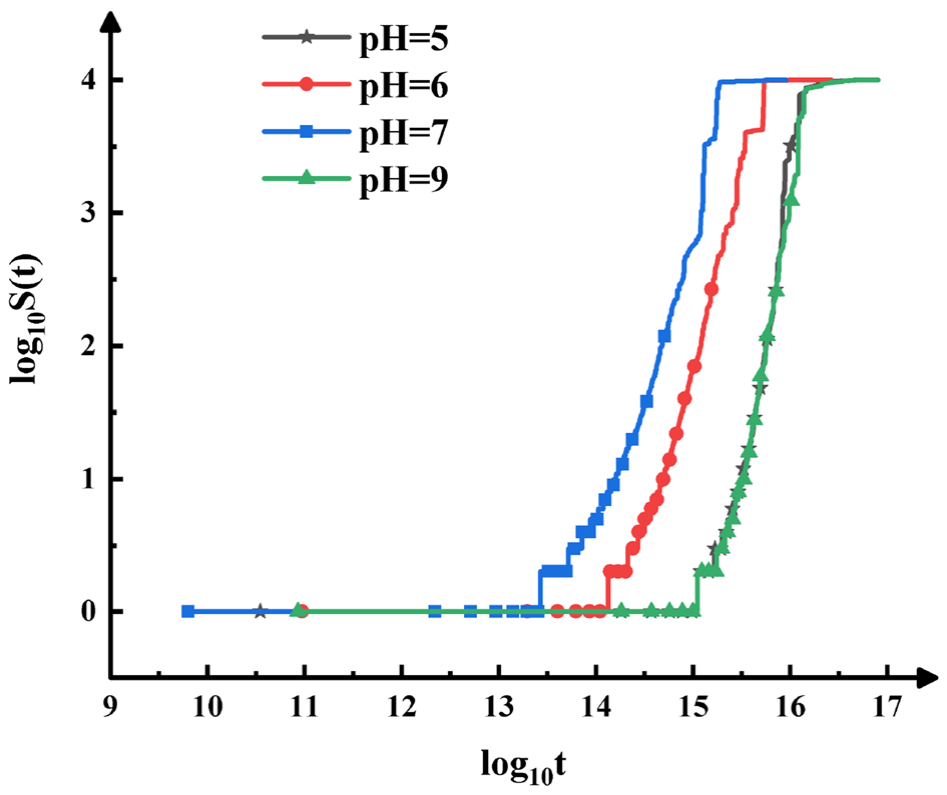
A log–log plot of the weighted average cluster size, *S*(*t*), as a function of time *t*, for the initial particle concentration, *c*, is 0.01 and different pH.

The changes of the weighted average cluster size with time under different pH conditions are shown in Fig. 6. When pH is equal to 7, the weighted average cluster size increases fastest and reaches the stable state first. The changes of the weighted average cluster size at pH = 5 and pH = 9 are almost the same, and the change speed is the slowest. Therefore, it can be concluded that when pH value is equal to PZC, the rapid aggregation occurs between the particles, with the highest aggregation rate and the most open cluster structure; when the difference between pH and PZC is larger, the sticking probability between particles is smaller, the aggregation rate is also smaller, and the cluster structure is more compact.

## Conclusions

By employing the theory that the aggregation of the variably charged particles depends on pH under given temperature, electrolyte concentration, and electrolyte type, this paper proposed an extended model based on the classic 3D on-lattice CCA model. In the extended CCA model, the sticking probability was set as a function of pH and PZC, and the change of pH was set as a time-dependent parameter. Through this extended CCA model, this research successfully verified that the aggregation rate of variably charged particles with pH changes conforms to Gaussian distribution, which is consistent with the results obtained by traditional experimental methods. And this model can be used to calculate the aggregation rate and the fractal dimension at different pH, and the stability pH interval of variably charged nanoparticles, as well as to predict the trend of the aggregation rate of different types of variably charged nanoparticles with pH. Besides, these simulation results indicated that the closer the pH is to PZC, the tighter the cluster structure is. And the greater the initial concentration of particles, the greater the rate of aggregation. This paper is a useful attempt and supplement to the study of the aggregation process of nanoparticles and provides a new method and idea for studying the effect of pH on the aggregation of nanoparticles. The extended model could provide new technical support for related scholars to study the aggregation of nanoparticles.
